# Anti‐Lung Cancer Effects and Related Mechanisms of α‐Caryophyllene In Vitro and In Vivo

**DOI:** 10.1002/fsn3.70862

**Published:** 2025-08-27

**Authors:** Lu Jin, Yuanquan Ran, Nian Yang, Lanlan Yang, Xiaoyan Jia, Huan Zhao, Qiong Hu, Bing Yang, Dongxin Tang, Minyi Tian

**Affiliations:** ^1^ National & Local Joint Engineering Research Center for the Exploitation of Homology Resources of Southwest Medicine and Food Guizhou University Guiyang China; ^2^ First Affiliated Hospital of Guizhou University of Traditional Chinese Medicine Guiyang China

**Keywords:** α‐caryophyllene, apoptosis, lung cancer, migration and invasion, nude mice, proliferation

## Abstract

α‐Caryophyllene is a natural condiment and flavoring additive. Herein, we first study the in vivo and in vitro anti‐lung cancer efficacy of α‐caryophyllene and its potential mechanism. In antitumor activity in vitro, α‐caryophyllene exhibited obvious selective cytotoxicity, and its cytotoxicity against lung cancer A549 cells (IC_50_ = 22.94 ± 0.56) was superior to the elemene injection (EI, positive control), while its toxicity against normal cell lines was lower (MRC‐5: IC_50_ = 76.66 ± 2.60 μg/mL, L929: IC_50_ = 44.04 ± 0.72 μg/mL). α‐Caryophyllene blocked the cell cycle in the G1 phase via reducing cyclin E2‐CDK2 and cyclin D3‐CDK 4/6 complexes and increasing p21, thereby suppressing A549 cell proliferation. Simultaneously, α‐caryophyllene activated the mitochondrial apoptotic pathway through elevating the Bax/Bcl‐2 ratio, lowering ΔΨm, releasing Cyt c, activating caspase 9 and caspase 3, and triggering PARP fragmentation. In addition, α‐caryophyllene downregulated N‐cadherin and MMP‐2 levels, thereby preventing the metastasis of A549 cells. In antitumor activity in vivo, α‐caryophyllene effectively suppressed transplanted tumor growth by inducing apoptosis of transplanted tumor cells and was superior to the positive control EI. Therefore, α‐caryophyllene has outstanding anticancer properties in vitro and in vivo, showing great potential to become a new anticancer drug in the pharmaceutical field.

## Graphical abstract

α‐Caryophyllene is a natural condiment and flavoring additive. In this study, α‐caryophyllene suppressed the proliferation of A549 cells, induced apoptosis via the mitochondrial pathway, and restricted cell metastasis in vitro. Besides, it repressed the growth of A549 transplantation tumors by causing apoptosis of tumor cells in vivo.

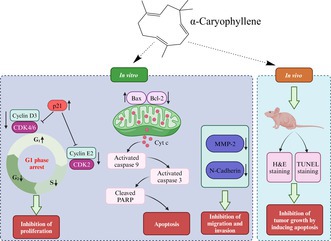

## 1 Introduction

In accordance with Global Cancer Statistics 2022, deaths caused by cancer account for about 1/6 of global deaths, among which lung cancer is the most deadly cancer worldwide, representing 18.7% of total deaths from all cancers (Bray et al. 2024). Non‐small cell lung cancer constitutes 80%–85% of all lung cancer (Chen et al. 2022). Multiple drugs derived from natural resources have been utilized for lung cancer therapy, such as paclitaxel, vincristine, etoposide, and irinotecan (Wang et al. 2021; Huang et al. 2012). Arguably, natural products are an important source for the discovery and development of new anticancer drugs.

Sesquiterpenes are naturally occurring terpenes composed of three isoprene units with a C15 skeleton (Frey 2020). Sesquiterpenes are widely present in essential oils and are their main pharmacologically active ingredients with anticancer activity (Huang et al. 2012; Durant et al. 2014; Spyridopoulou et al. 2017). For example, elemene, a sesquiterpene extracted from *Curcuma wenyujin* essential oil, has been exploited as a clinically used drug for the treatment of lung cancer in China, such as elemene injection (EI) and oral emulsion (Zhai et al. 2018; Zheng et al. 2018). Therefore, we screened the sesquiterpenes from essential oils by detecting their anti‐lung cancer effects using EI as a positive control drug, and our attention was attracted by α‐caryophyllene.

α‐Caryophyllene, also called humulene or α‐humulene, is a naturally occurring monocyclic sesquiterpene, which is composed of an 11‐membered ring with three E‐conjugated double bonds (Chen et al. 2019; de Lacerda Leite et al. 2021). α‐Caryophyllene was initially identified in the *Humulus lupulus* (hops) essential oil, from which it got its name (de Lacerda Leite et al. 2021). It is a natural food additive used as a condiment and flavoring additive in the food industry, especially in beer and some flavored foods, to confer food a distinctive aroma and flavor. Moreover, α‐caryophyllene is utilized in perfumes and cosmetics. Additionally, α‐caryophyllene is also widely found in various edible plants in nature, such as *Zingiber zerumbet* , *Myrica rubra* , and *Piper nigrum* (Alwakil et al. 2022; Hanušová et al. 2015; Bhatia et al. 2024). Past studies have found that α‐caryophyllene triggered apoptosis and suppressed hepatocellular carcinoma cell proliferation in vitro and in vivo (Chen et al. 2019). α‐Caryophyllene showed significant cytotoxicity against lung cancer A549 cells and colorectal cancer HT‐29 and HCT‐116 cells (El Hadri et al. 2010; Su et al. 2015). Furthermore, in colorectal cancer SW‐620 and Caco‐2 cells, it enhanced the antiproliferative effects of oxaliplatin and 5‐fluorouracil (Ambrož et al. 2019).

Natural sesquiterpenoids have been exploited as anti‐lung cancer agents for clinical use. α‐Caryophyllene is a natural edible sesquiterpene compound utilized in the food industry and cosmetics. However, there are few studies on its anti‐lung cancer effects. Therefore, our research aims to explore the in vitro and in vivo anti‐lung cancer efficacy and related mechanisms of α‐caryophyllene.

## 2 Materials and Methods

### 2.1 Chemicals and Reagents

α‐Caryophyllene was bought from the Tokyo Chemical Industry (Tokyo, Japan). The apoptosis Hoechst staining kit, cell mitochondria isolation kit, and mitochondrial membrane potential test kit (JC‐1) were purchased from the Beyotime Institute of Biotechnology (Shanghai, China). The Super ECL detection reagent was provided by Yeasen Biotechnology Co. Ltd. (Shanghai, China). MultiSciences Biotech Co. Ltd. (Hangzhou, China) supplied the cell cycle staining kit and the annexin V‐APC/PI apoptosis kit. The caspase 3 and cytochrome c (Cyt c) were purchased from Proteintech Group Inc. (Wuhan, China). The remaining antibodies were provided by Cell Signaling Technology (Danvers, Massachusetts, USA). Aladdin Industrial Corporation (Shanghai, China) supplied cisplatin. The remaining reagents were provided by Solarbio Life Sciences (Beijing, China).

### 2.2 Cytotoxic Activity

Four cell lines were utilized in the MTT assay: A549 (non‐small cell lung adenocarcinoma cells), L929 (murine fibroblast cells), MRC‐5 (fetal lung fibroblast cells), and NCI‐H1299 (non‐small cell lung cancer cells). As a positive control drug, cisplatin and elemene emulsion injection (EI) were applied. After counting cells, 80 μL cell suspension was added to 96‐well plates (5000 cells/well) and incubated in an incubator (37°C, 5% CO_2_) for 24 h. Afterwards, different concentrations of α‐caryophyllene solutions (20 μL) were pipetted into the wells, and the final concentrations of α‐caryophyllene were 5, 10, 20, 40, 80, and 160 μg/mL. Subsequently, the cells were maintained in culture for 48 h. Next, MTT (12 μL, 5 mg/mL) was pipetted into each well of the 96‐well plates, and culturing was stopped after 4 h. The wells' supernatant was cautiously aspirated out using a syringe, and 150 μL of DMSO was added. The 96‐well plates were gently shaken (10 min) so that DMSO fully dissolved the formazan in the cells. Lastly, an Infinite M200 Pro microreader (Tecan, Switzerland) was employed to determine the absorbance value (490 nm). IC_50_ value was subsequently calculated on SPSS 26.0.

### 2.3 Clone Formation Assay

A549 cell suspension was adjusted to a concentration of 100 cells/mL. Next, 2 mL cell suspension was transferred to a 6‐well plate and cultured for 24 h. The original medium was discarded, and the culture was continued by adding α‐caryophyllene solution (0, 20, 30, and 40 μg/mL). After 48 h incubation, the old culture medium was pipetted out, and a fresh medium without α‐caryophyllene solution was added to continue the culture. The formation of cell colonies was observed daily, and cells were cultured until visible white spots appeared on the bottom of the 6‐well plate. After removing the culture medium, the cells underwent two PBS washes and fixation using 800 μL of 4% paraformaldehyde fixative for 30 min. After discarding the fixative, 800 μL of 0.1% crystal violet dye was added, and the staining was performed in the dark for 15 min. Afterwards, we discarded the dye solution, gently washed away the remaining dye solution with distilled water, and placed the 6‐well plate at room temperature to allow the water to evaporate. Then, we took pictures, counted the number of clone formations, and calculated the clone formation rate using the following formula:

$$\text{Clone formation rate}=\frac{\text{Quantity of formed cell clones}\;}{\text{Quantity of inoculated cells}\;}\times 100\%$$

### 2.4 Cell Cycle Assay

In each well of a 6‐well plate, 3 × 10^5^ cells were added and cultured for 24 h. Subsequently, α‐caryophyllene solutions with concentrations of 20, 40, 60, and 80 μg/mL were administered. Following 48 h of cultivation, PBS was used to wash the cells. Following the instructions in the cell cycle staining solution kit, we added DNA staining solution (1 mL) to each well. Next, the cells were left to incubate away from light for 30 min. Then, flow cytometry (ACEA Biosciences, San Diego, CA, USA) was applied to determine the distribution of the cell cycle.

### 2.5 Apoptosis Detection

#### 2.5.1 Morphology Test

For morphological observation, A549 cell suspension was inoculated into a 6‐well plate (5 × 10^5^ cells/well) and cultured for 24 h. Next, different doses of α‐caryophyllene solution (0, 20, 40, 60, and 80 μg/mL) were employed to treat the cells for 48 h. At the end of cultivation, we used a Leica DMi8 microscope (Leica Microsystems, Germany) to view the morphological changes.

For AO/EB detection, the A549 cell line was processed in the same way as above. After 48 h of cultivation, we discarded the culture medium and performed rinsing twice with PBS. Then, we mixed the AO stain (100 μg/mL) and EB stain (100 μg/mL) in equal proportions and placed 800 μL of the AO/EB mixture into each well. The staining was applied in the dark for 5 min. Lastly, we used a Leica DMi8 fluorescence microscope to observe and take pictures.

For Hoechst 33258 test, after treatment consistent with morphological observations, cells underwent two PBS washes and fixation for 20 min using 500 μL of fixative (4% paraformaldehyde). We rinsed the fixative with PBS and stained to avoid light for 5 min using Hoechst 33258 staining solution. Following the dye solution was discarded, the PBS was used twice to wash the cells, and changes in cell nucleus morphology were viewed.

#### 2.5.2 Annexin V‐APC/PI Assay

In 6‐well plates, A549 cells at a cell density of 3 × 10^5^/well were cultivated for 24 h. Next, the cells were exposed to different concentrations (0, 20, 40, 60, and 80 μg/mL) of α‐caryophyllene solution for 24 h of cultivation. Afterward, the α‐caryophyllene‐treated A549 cell line was rinsed with pre‐cooled PBS, collected into centrifuge tubes for centrifugation (800 rpm, 5 min), and resuspended in 1× Binding Buffer (500 μL). Subsequently, we added 10 μL PI and 5 μL Annexin V‐APC, vortexed gently, and cultured for 5 min at room temperature, avoiding light. Finally, we performed flow cytometry to ascertain cell apoptosis.

### 2.6 JC‐1 Staining

In 6‐well plates, A549 cells were inoculated at a density of 5 × 10^5^/well and cultured for 24 h and then treated with varying doses of α‐caryophyllene solution (0, 10, 20, 40, 80, and 160 μg/mL) for 48 h. Next, we removed the culture medium and rinsed the cells with PBS. Afterward, 900 μL of JC‐1 staining working solution and 900 μL of culture medium were pipetted into each well, thoroughly mixed, and cultured in the incubator for 20 min. After washing the cells twice with JC‐1 staining solution (1×), each well received 2 mL of culture medium. Finally, the cells were viewed and photographed using an inverted fluorescence microscope.

### 2.7 Cell Metastasis Detection

#### 2.7.1 Wound Healing Test

In 6‐well plates, A549 cells (5 × 10^5^ cells per well) were inoculated until the cells became a monolayer of confluent cells. Subsequently, the bottom of the well was scratched vertically with a 200 μL pipette tip. Next, we discarded the culture medium and rinsed away the floating cells with PBS. Afterwards, α‐caryophyllene solution (0, 10, 20, and 30 μg/mL) prepared in low‐serum culture medium (0.05% FBS) was pipetted into the wells, and the scratch state at 0 h was recorded under a Leica DMi8 microscope. After 48 h incubation, we observed cell migration and took photographs. The scratch areas at 0 and 48 h were recorded utilizing Leica Application Suite software (version 4.12.0). The migration rate is calculated as follows:

$$\text{Migration rate}=\frac{S_{\text{scratch area of}\;0\;h}-S_{\text{scratch area of}\;48\;h}}{S_{\text{scratch area of}\;0\;h}}\times 100\%$$

#### 2.7.2 Transwell Invasion Detection

In Transwell invasion detection, we used Corning BioCoat Matrigel invasion chambers (New York, USA). In the lower chamber, 750 μL of α‐caryophyllene solution (0, 10, 20, and 40 μg/mL) diluted with medium containing 15% FBS was added. Next, A549 cells were digested, collected, and resuspended in a medium containing 5% FBS. Subsequently, we pipetted the cell suspension (250 μL, 1 × 10^5^ cells/well) into the upper chamber and simultaneously added 250 μL of α‐caryophyllene solution (diluted with culture medium containing 5% FBS), ensuring the α‐caryophyllene concentration was the same in both the upper and lower chambers. The upper chamber was gently placed into the lower chamber to avoid air bubbles between the upper and lower chambers and incubated in an incubator for 48 h. After the incubation ended, the upper chamber was taken out, the medium was pipetted out, and PBS was employed twice to wash the chamber. Next, we fixed the cells in the upper chamber with 4% paraformaldehyde for 2 min, followed by removal of the fixative and two PBS washes. Subsequently, we incubated the upper chamber with anhydrous methanol for 20 min, removed the methanol, and washed the chamber with PBS. The cells were stained with 0.1% crystal violet for 15 min and washed twice with PBS. Afterward, the residual cells on the upper chamber's upper surface were gently wiped using a cotton swab. After air‐drying, random fields of the lower surface of the upper chamber were photographed using a microscope, and the invaded cells were counted.

### 2.8 Western Blotting Assay

In 6‐well plates, A549 cells (5 × 10^5^ cells/well) were cultured for 24 h and exposed to varying α‐caryophyllene concentrations (0, 20, 40, and 60 μg/mL) for 48 h. Subsequently, A549 cells and culture medium were collected into the centrifuge tube. After centrifugation (10,000 rpm, 4 min), the supernatant was discarded. Next, lysis buffer was added to the centrifuge tube, and the cells were placed on ice to lyse for 30 min. After centrifugation (12,000 rpm, 10 min), the supernatant was harvested. The total cellular protein was supplemented with the loading buffer, heated in a metal bath (100°C) for 5 min, and stored in a −80°C freezer. A cell mitochondria isolation kit was employed to extract cytoplasmic and mitochondrial proteins. The concentration of the extracted proteins was ascertained according to the BCA assay kit. The 10% SDS‐PAGE was used for protein separation (120 V, 60 min), and then the separated proteins were transferred to the PVDF membrane (90 V, 60 min). Next, we washed the PVDF membrane for 5 min with TBS‐T (TBS, 0.1% Tween‐20). After removing the TBST, we placed the PVDF membrane in a blocking solution for 1 h. Then, the primary antibody was added and incubated overnight at 4°C. Next, we collected the primary antibody, washed the PVDF membrane with TBST (3 times, 5 min), and added the secondary antibody. Following incubation for 1 h, the PVDF membrane was rinsed with TBST (3 times, 5 min), and the Super ECL detection reagent was added. The image was captured using the ChemiDoc touch imaging system.

### 2.9 Nude Mouse Transplanted Tumor

Laboratory Animal Ethics Committee of Guizhou University approved this animal study with ethics approval number: EAE‐GZU‐2023‐E051. The experimental animals were SPF‐grade athymic BALB/c‐nu nude mice (half male and half female, 5 weeks old) supplied by SiPeiFu Biological Co. Ltd. (Beijing, China). The nude mice were adaptively fed for 1 week. Subsequently, the right forelimb armpit of the nude mice was inoculated with 200 μL of A549 cell solution (2 × 10^7^ cells/mL) via subcutaneous injection. When the tumor grew to 100 mm^3^, the nude mice were randomly assigned into 3 groups (*n* = 10 in each group). Elemene injection (100 mg/kg) and α‐caryophyllene (50 mg/kg) were administered on alternate days via intraperitoneal injection for 21 days. At the same time, the tumor size of the nude mice was recorded. Tumor volume was calculated as: tumor volume = (longest diameter × shortest diameter^2^)/2. After stopping the administration, the mice were anesthetized with ether and sacrificed via cervical dislocation. We stripped the transplanted tumors, obtained their weight (W), and calculated the tumor inhibition rate using the following formula:

$$\text{Tumor inhibition rate}=\frac{W_{\text{model group}}-W_{\text{drug administration group}}}{W_{\text{model group}}}\times 100\%$$

### 2.10 Histological Examination of Transplanted Tumor

The transplanted tumors were fixed with tissue fixative (4% paraformaldehyde) for 24 h, followed by gradient dehydration with alcohol, and then immersed in wax. The wax‐soaked tissue was embedded in an embedding machine and prepared into 4 μm thick tissue sections. The tissue sections were floated in warm water (40°C) in a spreading machine to flatten the tissue. Next, we collected the tissue sections and baked them in an oven (60°C). Afterward, we stored the tissue sections at room temperature for further H&E and TUNEL staining assays.

#### 2.10.1 H&E Staining

We dewaxed the tissue sections and washed them with distilled water. Then, the sections were stained with a hematoxylin staining solution for 5 min and rinsed with water. After that, the sections were differentiated with a hematoxylin differentiation solution, treated with a hematoxylin bluing solution, and washed again with distilled water. Next, the sections were sequentially dehydrated for 5 min in 85% and 95% alcohol and were stained for 5 min in an eosin staining solution. Then, the sections were dehydrated, processed with xylene, and sealed with neutral gum. A Panoramic 250/MIDI scanner was used to observe the sections, and collected images were analyzed with a 3DHistech CaseViewer 2.4 program (Budapest, Hungary).

#### 2.10.2 TUNEL Staining

The tissue sections were subjected to a dewaxing process and washed with water. Subsequently, the sections were incubated in protease K solution (37°C, 22 min), placed in PBS, and washed on a decolorization shaker (3 times, 5 min). After the sections were dried, permeabilize working solution (0.1% triton) was added dropwise to cover the sections. The sections were incubated for 20 min at room temperature and washed with PBS (3 times, 5 min). Then, the TUNEL reaction solution was pipetted into the tissue sections dropwise and incubated for 1 h at 37°C. The sections were again washed with PBS (3 times, 5 min). Next, we removed the PBS, added DAPI solution to the sections, and placed the sections at room temperature for 10 min in the dark. Following PBS washing of the sections, excess liquid was removed with absorbent paper, and an anti‐fade mounting medium was added dropwise to mount the sections. Lastly, the sections were viewed using a Panoramic 250/MIDI scanner, and images were captured and analyzed.

### 2.11 Statistical Analysis

In this study, the means ± standard deviation (SD) represents the data. Statistical analysis was performed using SPSS 26.0 (SPSS Inc., Chicago, IL, USA). The two‐tailed unpaired *t*‐test assessed significant differences in tumor inhibition rate between the α‐caryophyllene and the EI groups. In the remaining data analyses, one‐way analysis of variance (ANOVA) with the least significant difference (LSD) was employed to detect significant differences. The *p* values < 0.05 were considered statistically significant (*p < 0.05, **p < 0.01, ***p < 0.001).

## 3 Results

### 3.1 Cytotoxicity of α‐Caryophyllene

**FIGURE 1:**
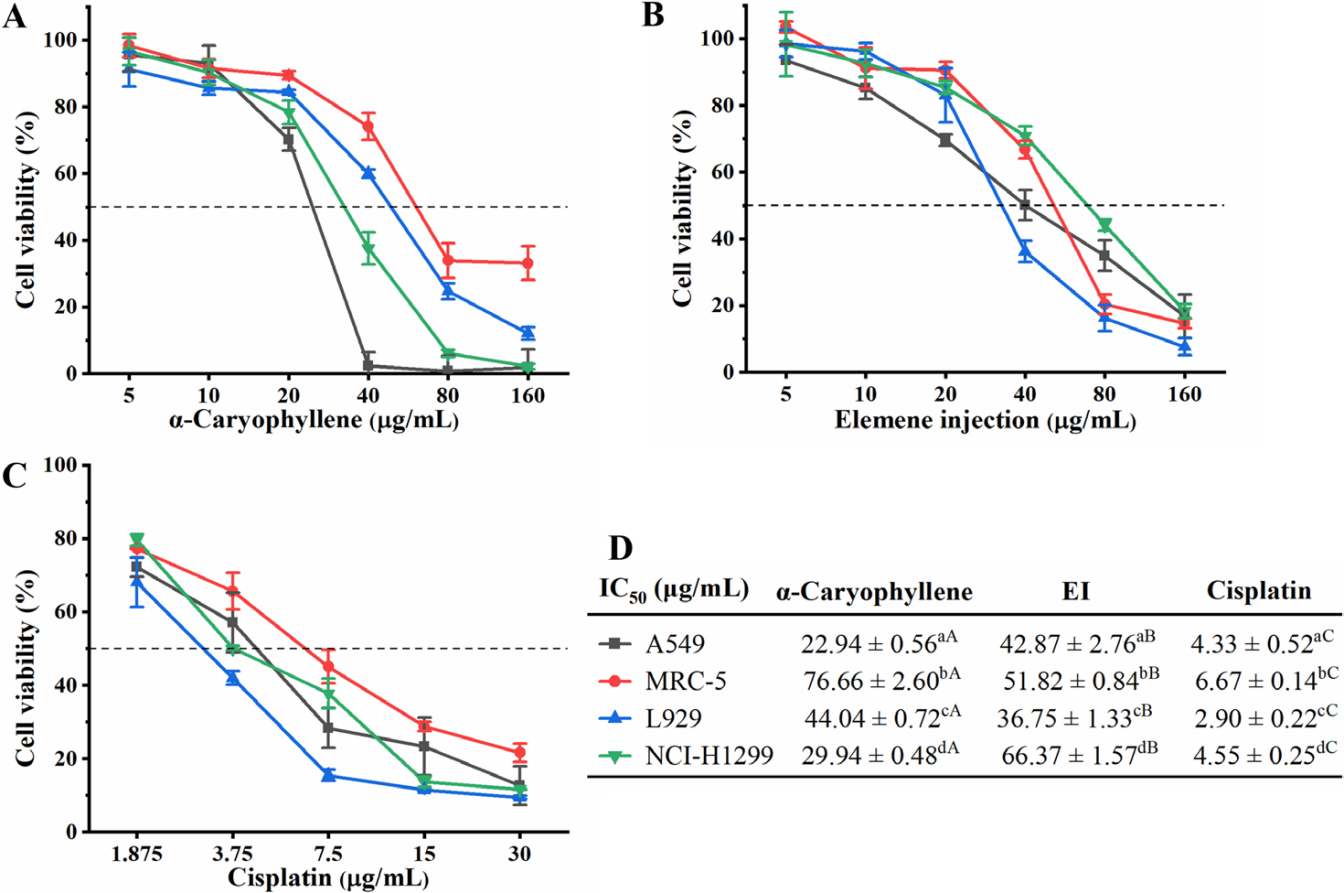
Cytotoxic activities of α‐caryophyllene (A), elemene injection (B), and cisplatin (C) on the NCI‐H1299, MRC‐5, A549, and L929 cell lines. Elemene injection (EI) and cisplatin were positive control drugs. (D) IC_50_ values of α‐caryophyllene, EI, and cisplatin against the four cell lines. IC_50_: Half inhibitory concentration. Significant differences (*p* < 0.05) are indicated by different letters in the same row (A–C) and the same column (a–d).

MTT assay was utilized to evaluate the cytotoxic activity of α‐caryophyllene on cancer cells NCI‐H1299 and A549 as well as normal cells MRC‐5 and L929. Cisplatin and elemene injection (EI) were employed as the positive controls. As displayed in Figure 1, α‐caryophyllene showed remarkable cytotoxicity against two cancer cell lines with IC_50_ values of 22.94 ± 0.56 μg/mL (A549) and 29.94 ± 0.48 μg/mL (NCI‐H1299). Compared to cancerous cell lines, α‐caryophyllene showed weak cytotoxic activity against noncancerous cell lines with IC_50_ values of 44.04 ± 0.72 μg/mL (L929) and 76.66 ± 2.60 μg/mL (MRC‐5). It is noteworthy that α‐caryophyllene was more cytotoxic to A549 cells than the positive control EI (IC_50_ = 42.87 ± 2.67 μg/mL). All the data indicated that α‐caryophyllene displayed selective cytotoxicity against cancer cells, particularly in A549 cells. Therefore, we selected A549 cells for the follow‐up study on the anticancer effects of α‐caryophyllene.

### 3.2 α‐Caryophyllene's Inhibitory Effects on Clone Formation

**FIGURE 2:**
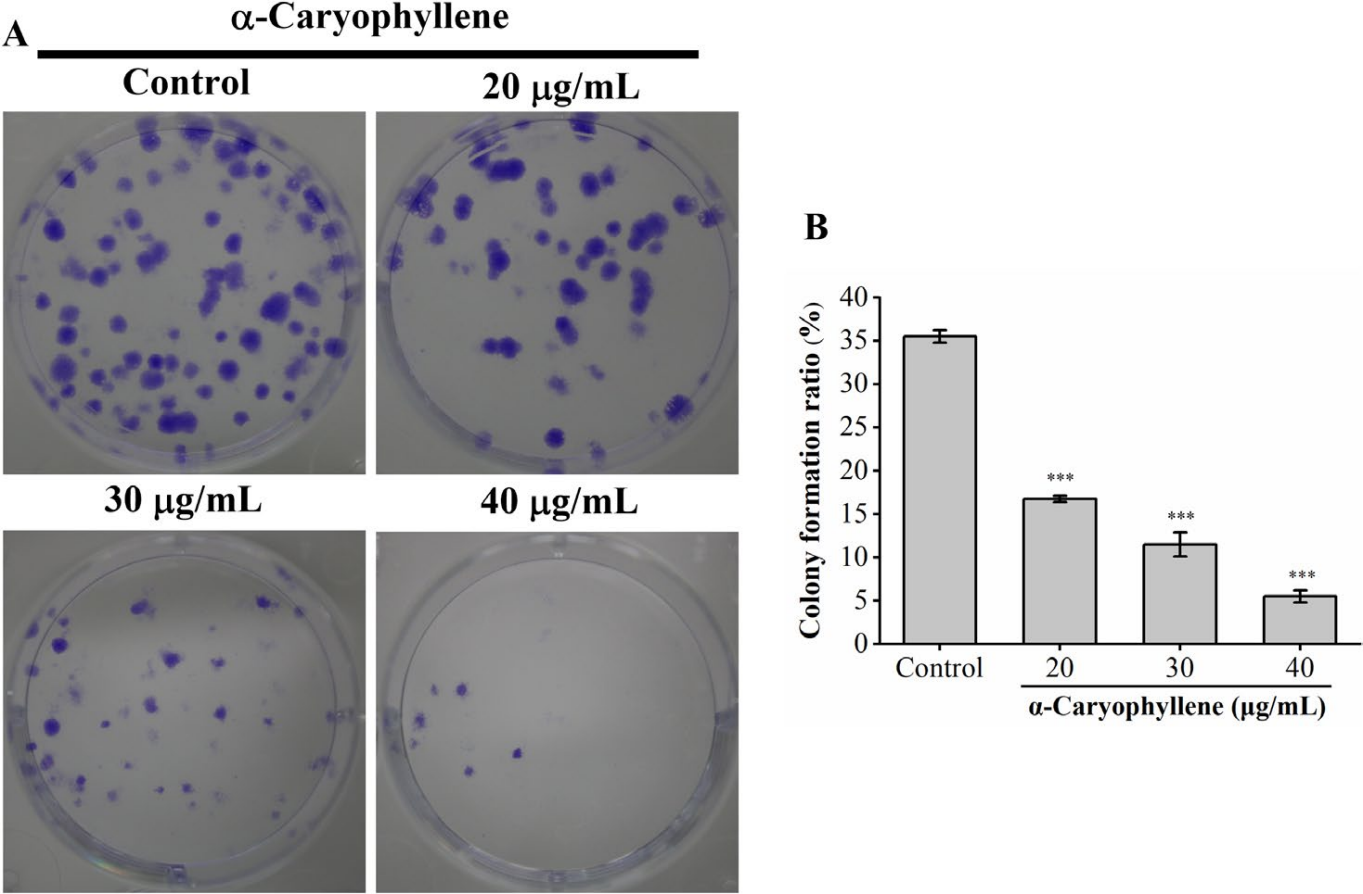
α‐Caryophyllene's impact on the clone‐forming ability of A549 cells. A549 cells (200 cells/well) were seeded in 6‐well plates containing α‐caryophyllene to detect its proliferation inhibitory effects. (A) The representative images of A549 colonies dyed with crystal violet. (B) Clone formation rate (%) of A549 cells. Compared to the control group, ***p < 0.001.

The clone‐forming ability of A549 cells exposed to different doses of α‐caryophyllene was tested to evaluate its impact on cell proliferation. The findings revealed that α‐caryophyllene considerably decreased the quantity and size of A549 cell colonies. As shown in Figure 2, in contrast to the control group (35.50% ± 0.71%), after treating A549 cells with α‐caryophyllene at doses of 20, 30, and 40 μg/mL, the clone formation rate was dramatically lowered to 16.75% ± 0.35%, 11.50% ± 1.41%, and 5.50% ± 0.71%, respectively. The above findings suggested that α‐caryophyllene effectively and dose‐dependently suppressed A549 cell proliferation.

### 3.3 α‐Caryophyllene Induced Cell Cycle Arrest at the G1 Phase

**FIGURE 3:**
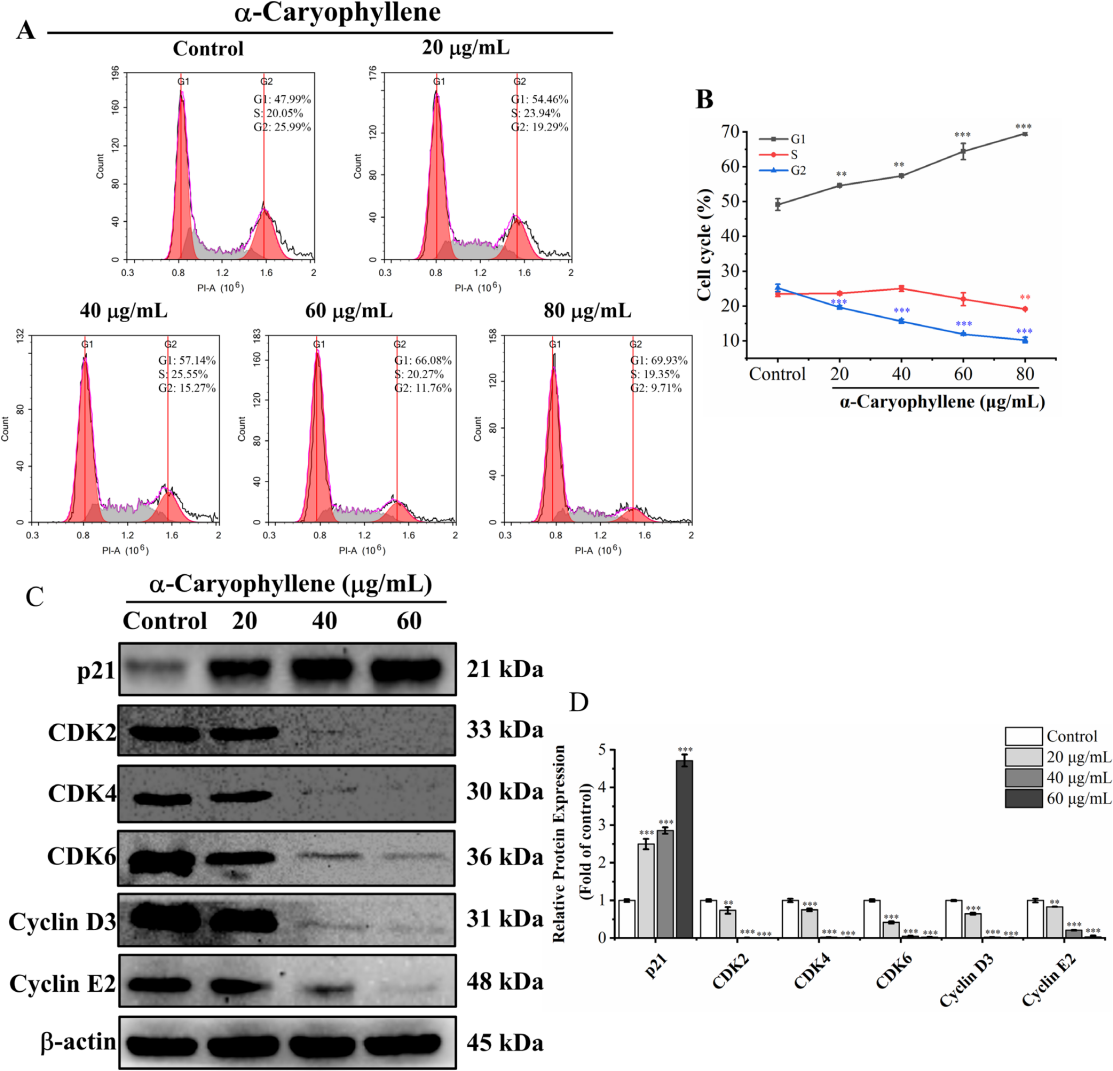
α‐Caryophyllene arrested the cell cycle in the G1 phase. (A) The cell cycle profile of A549 cells exposed to α‐caryophyllene was ascertained through flow cytometry. (B) The G1, S, and G2 phase percentages of A549 cells. (C) Western blot was used to detect cyclin E2, CDK4, CDK2, CDK6, cyclin D3, and p21 levels in A549 cells treated with α‐caryophyllene. (D) The relative expression levels of the cyclin E2, CDK4, and p21 proteins. In contrast to the control group, **p < 0.01, ***p < 0.001.

In order to determine whether α‐caryophyllene's antiproliferative effect was caused by the cell cycle block, we utilized flow cytometry to analyze the cell cycle profile. As evidenced by the flow cytometry results (Figure 3A,B), after A549 cells were exposed to α‐caryophyllene at doses of 10, 20, 40, 80, and 160 μg/mL, the proportion of cells in the G1 phase significantly raised from 49.16% ± 1.65% in the control group to 54.61% ± 0.21%, 57.38% ± 0.33%, 64.42% ± 2.35%, and 69.59% ± 0.49%, respectively. In addition, the proportion of cells in the G2 phase was significantly reduced, and the proportion of cells in the S phase was reduced at 80 μg/mL. The findings indicated that α‐caryophyllene suppressed cell proliferation by arresting the cell cycle of A549 cells in the G1 phase.

Western blot was employed to detect the levels of G1 phase regulatory proteins in A549 cells, and the findings were presented in Figure 3C,D. In contrast to the control group, α‐caryophyllene markedly increased the level of p21 and significantly decreased the level of cyclin E2, CDK4, CDK2, CDK6, and cyclin D3 in a concentration‐dependent manner. Taken together, this evidence demonstrated that α‐caryophyllene impeded the proliferation of A549 cells by inducing G1 phase arrest.

### 3.4 α‐Caryophyllene Induced Apoptosis of A549 Cells

**FIGURE 4:**
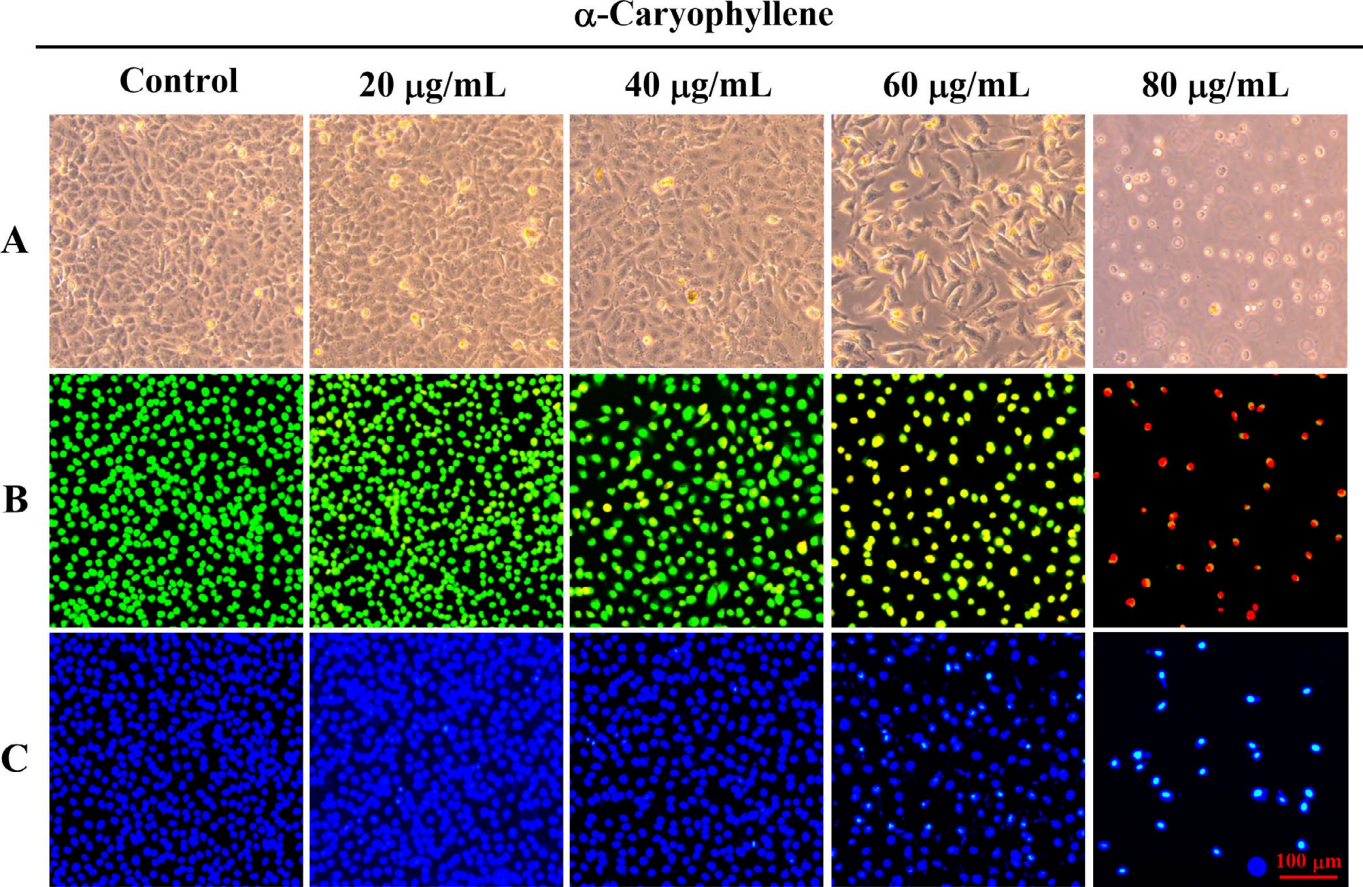
The changes in A549 cell morphology after exposure to different doses of α‐caryophyllene. (A) Morphological changes of A549 cells under a phase contrast microscope. (B, C) After staining with AO/EB (B) and Hoechst 33258 (C), the morphological changes of nuclei were visualized via an inverted fluorescence microscope.

Tumors differ from normal cells in that they evade cell death, and inducing apoptosis has become an important means of treating tumors (Yang et al. 2024). Therefore, we investigated the effect of α‐caryophyllene on inducing apoptosis in cancer cells. Morphological observations indicated that A549 cells were exposed to α‐caryophyllene, showing typical features of apoptosis: cell rounding and shrinking (Figure 4A). As illustrated in Figure 4B, the results of AO/EB staining displayed that the percentage of orange‐red nuclei gradually increased with the ascending concentration of α‐caryophyllene, indicating a gradual increase in apoptotic cells. The Hoechst 33258 staining findings exhibited that following α‐caryophyllene treatment, the percentage of bright blue fluorescent cells with dense nuclei gradually elevated, showing the characteristics of apoptosis (Figure 4C).

**FIGURE 5:**
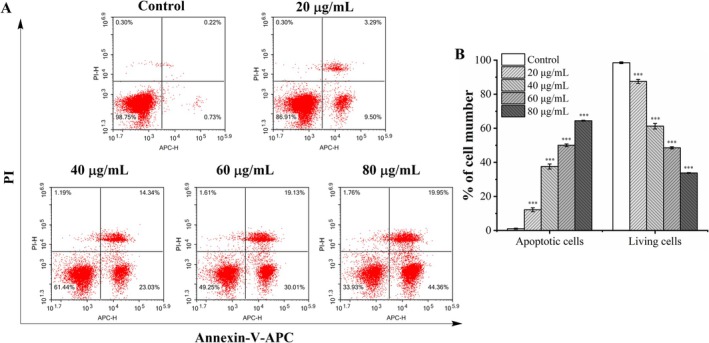
α‐Caryophyllene induced apoptosis was quantitatively analyzed by flow cytometry. (A) A549 cells were treated with the specified doses of α‐caryophyllene, labeled with PI and Annexin V‐APC, and analyzed employing a flow cytometer. Live cells: Lower left quadrant (APC–/PI–); necrotic cells: Upper left quadrant (APC–/PI+); early apoptotic cells: Lower right quadrant (APC+/PI–); late apoptotic cells: Upper right quadrant (APC+/PI+). (B) The percentage of apoptotic and living cells. In contrast to the control group, ***p < 0.001.

The Annexin V‐APC/PI staining was used in flow cytometry to quantify α‐caryophyllene induced apoptosis (Figure 5). Compared with the control group (2.32% ± 0.15%), the apoptosis rates of A549 cells observably raised to 8.63% ± 0.36%, 18.12% ± 0.64%, 23.93% ± 1.28%, and 30.64% ± 1.61% after exposure to α‐caryophyllene at doses of 20, 40, 60, and 80 μg/mL, respectively. The above findings illustrated that α‐caryophyllene dose‐dependently triggered apoptosis in A549 cells.

### 3.5 α‐Caryophyllene Reduced Mitochondrial Membrane Potential

**FIGURE 6:**
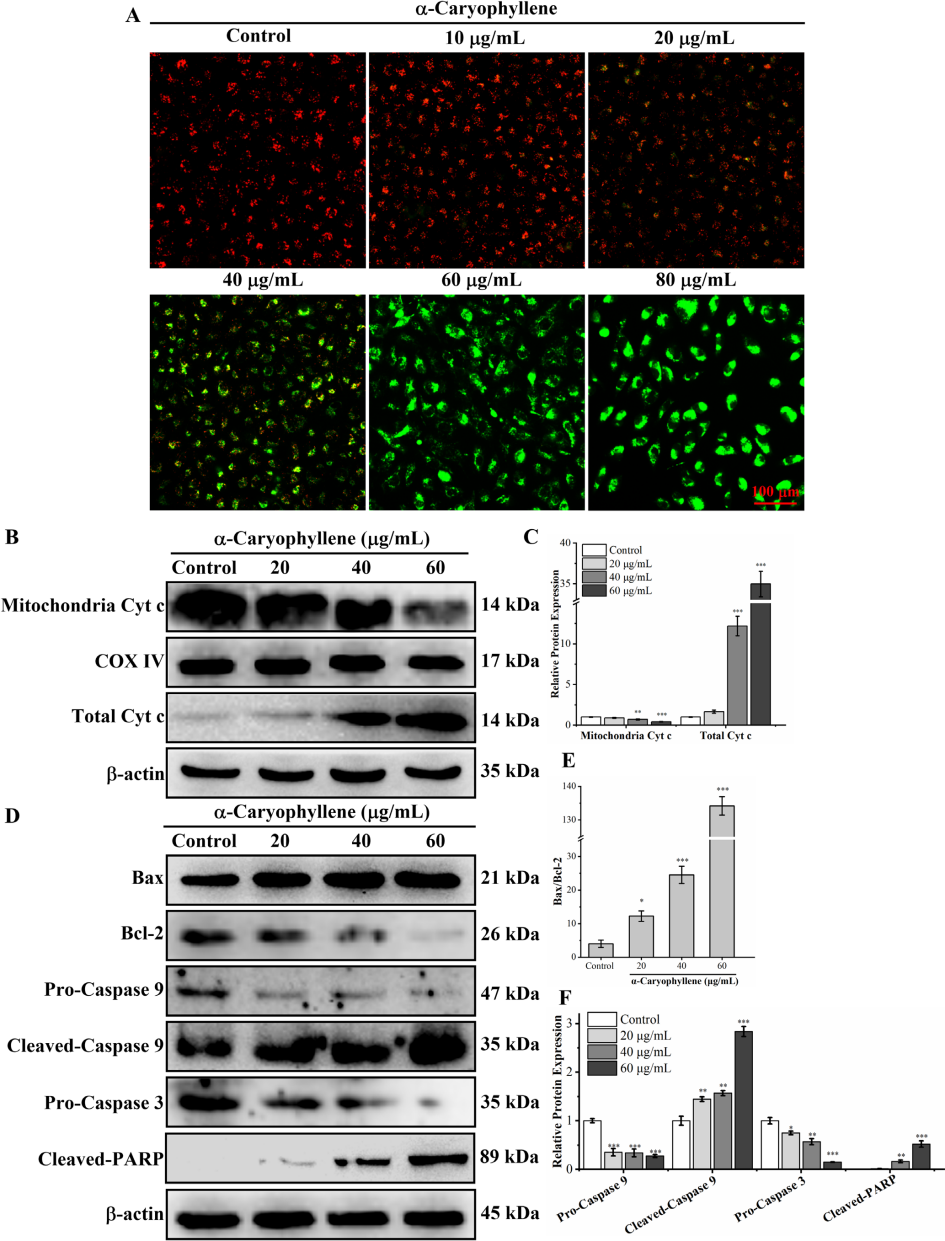
α‐Caryophyllene triggered mitochondria‐mediated apoptosis in A549 cells. (A) An inverted fluorescent microscope was employed to measure the ΔΨm in α‐caryophyllene‐treated A549 cells labeled with JC‐1. (B, C) Western blot analysis was performed to determine the mitochondrial Cyt c and total Cyt c levels in A549 cells treated with α‐caryophyllene. (D–F) Western blot analysis was utilized to detect the levels of cleaved‐PARP, pro‐caspase 9, Bcl‐2, pro‐caspase 3, Bax, and cleaved‐caspase 9 proteins. Compared to the control group, *p < 0.05, **p < 0.01, ***p < 0.001.

The reduction in mitochondrial membrane potential (ΔΨm) is a crucial event in the early stages of apoptosis (Yang et al. 2024). Therefore, we detected ΔΨm using the JC‐1 fluorescent probe. In JC‐1 staining, red fluorescence is produced by JC‐1 aggregating to form polymers in the mitochondrial matrix of live cells. However, because of the decreased ΔΨm in apoptotic cells, JC‐1 only exists in the cytoplasm in a monomeric state, thereby emitting green fluorescence. As observed in Figure 6A, as the concentration of α‐caryophyllene increased, the percentage of cells displaying green fluorescence raised gradually, and the percentage of cells exhibiting red fluorescence decreased progressively. The aforementioned findings suggested that α‐caryophyllene reduced ΔΨm in A549 cells.

Western blot is used to examine critical proteins in the mitochondria‐mediated apoptosis pathway. As illustrated in Figure 6B,C, the levels of mitochondrial Cyt c were significantly decreased after treatment with α‐caryophyllene. In opposition to this, total Cyt c was raised considerably, suggesting that Cyt c was released from mitochondria. According to Figure 6D,E, α‐caryophyllene downregulated Bcl‐2 and upregulated Bax levels; therefore, the Bax/Bcl‐2 ratio was markedly enhanced. As indicated in Figure 6D,F, α‐caryophyllene increased cleaved‐caspase 9 and cleaved‐PARP levels and reduced pro‐caspase 9 and pro‐caspase 3 levels, suggesting that the caspase cascade is activated, leading to PARP cleavage. Therefore, α‐caryophyllene induced mitochondria‐mediated apoptosis in A549 cells.

### 3.6 α‐Caryophyllene Inhibited Metastasis of A549 Cells

**FIGURE 7:**
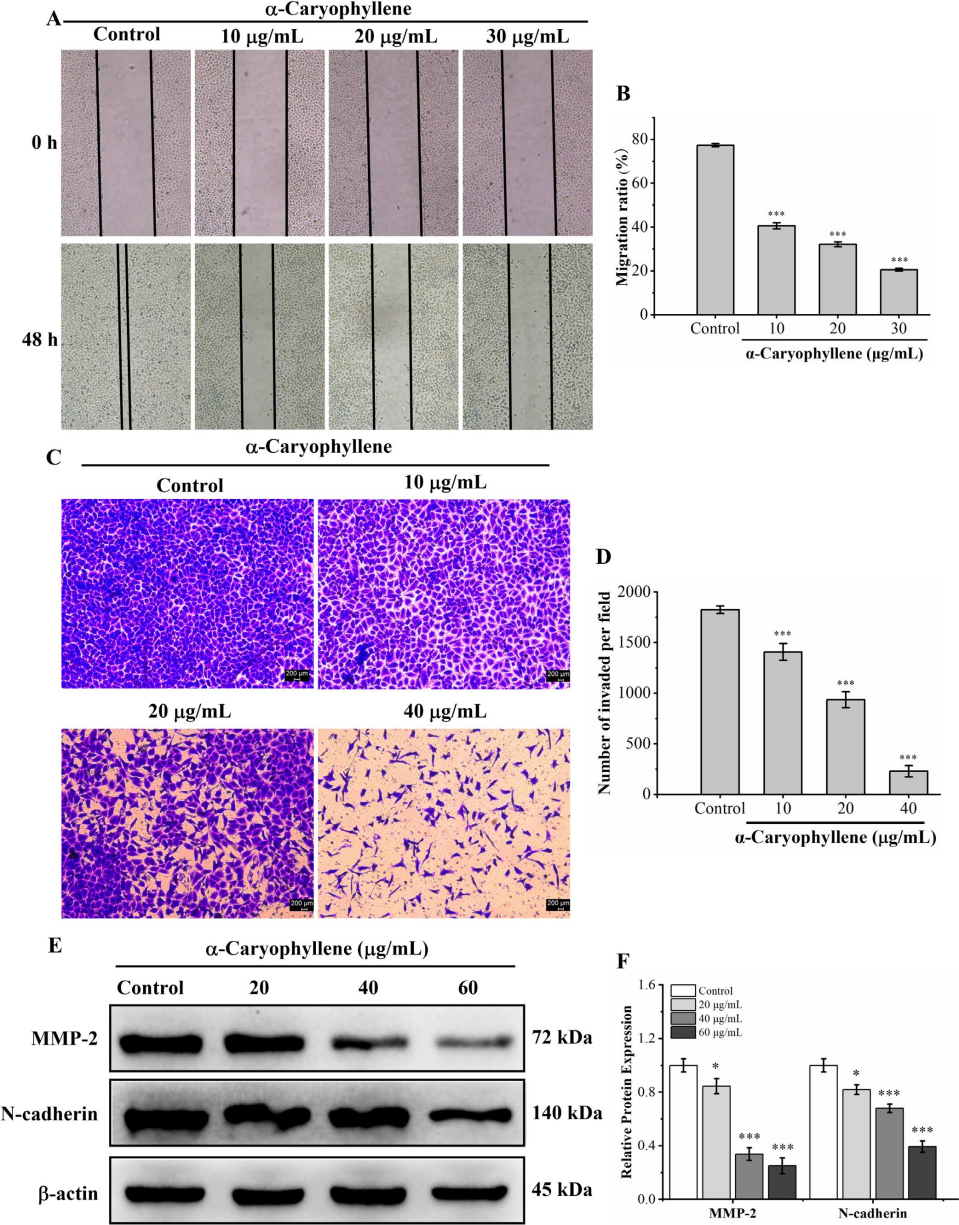
The influence of α‐caryophyllene on metastasis and expression of metastasis‐related proteins in A549 cells. (A) The impact of α‐caryophyllene on A549 cells' capacity to migrate was examined using the wound healing assay. (B) Employing the migration ratio (%) to provide a quantitative analysis of the migration capacity. (C, D) The invasion ability was measured and quantified by Transwell invasion assay. (E) Levels of N‐cadherin and MMP‐2 (metastasis‐associated proteins) in A549 cells following α‐caryophyllene treatment were detected by Western blot analysis. (F) The relative expression levels of MMP‐2 and N‐cadherin proteins. Compared with the control group, *p < 0.05, ***p < 0.001.

We applied wound healing and Transwell invasion assays to explore the influence of α‐caryophyllene on the migratory and invasive abilities of A549 cells. As observed in Figure 7A,B, in comparison with the control group (77.35% ± 0.72%), the migration rates of A549 cells treated with α‐caryophyllene at various doses (10, 20, 30 μg/mL) were substantially lower, which were 40.58% ± 1.41%, 32.16% ± 1.10%, and 20.62% ± 0.66%, respectively, indicating that α‐caryophyllene inhibited the lateral migration ability of A549 cells. The Transwell invasion experiment findings (Figure 7C,D) demonstrated that α‐caryophyllene considerably reduced the number of invaded A549 cells in a dose‐dependent manner compared with the control group, indicating that α‐caryophyllene inhibited the invasion ability of A549 cells. Western blotting was used to evaluate the impact of α‐caryophyllene on proteins associated with metastasis. As shown in Figure 7E,F, α‐caryophyllene significantly decreased the expression levels of N‐cadherin and MMP‐2. The above findings suggested that via downregulating the levels of N‐cadherin and MMP‐2, α‐caryophyllene inhibited the invasion and migration abilities of A549 cells. Therefore, α‐caryophyllene suppressed the metastatic ability of A549 cells in a concentration‐dependent manner.

### 3.7 Effect of α‐Caryophyllene on Transplanted Tumors in Nude Mice

**FIGURE 8:**
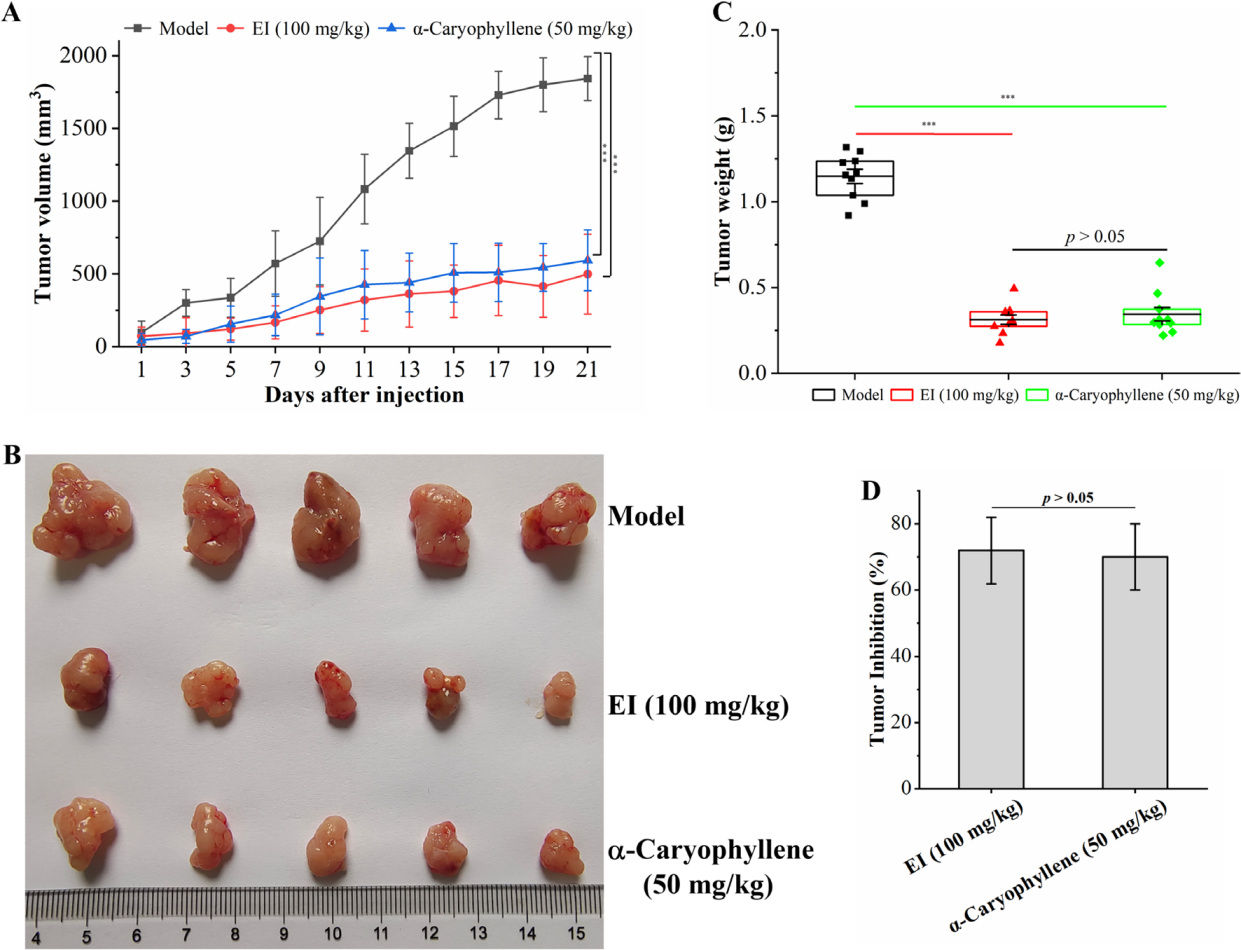
The in vivo antitumor effect of α‐caryophyllene in a nude mouse xenograft model. (A) α‐Caryophyllene's effect on tumor volume curve. (B) Representative images of transplanted tumors in each group at 21 d after administration. (C) α‐Caryophyllene's impact on transplanted tumor weight. (D) The inhibitory rate of α‐caryophyllene on transplanted tumors. Compared with the control group, ***p < 0.001.

In our study, we further used a nude mouse xenograft model to explore the antitumor effect of α‐caryophyllene in vivo. As illustrated in Figure 8A,B, after 21 days of drug administration, compared with the model group (1842.22 ± 151.47 mm^3^), the transplanted tumor volume treated with 50 mg/kg α‐caryophyllene and the positive control EI (100 mg/kg) was markedly suppressed, being 593.07 ± 209.48 mm^3^ and 413.94 ± 212.55 mm^3^ respectively. Besides, there was no obvious difference in tumor volume between the α‐caryophyllene group (50 mg/kg) and the EI group (100 mg/kg) (*p* > 0.05). As illustrated in Figure 8C,D, in comparison with the tumor weight of the model group (1.15 ± 0.13 g), the tumor weights of the EI group and the α‐caryophyllene group were remarkably reduced to 0.31 ± 0.09 g and 0.34 ± 0.13 g, respectively, and there was no significant difference in tumor weight between the α‐caryophyllene group and the EI group (*p* > 0.05). At the same time, the tumor inhibition rates of EI and α‐caryophyllene groups were 71.95% ± 10.05% and 69.97% ± 9.97%, respectively, with no statistically significant difference between the two groups (*p* > 0.05). The above results demonstrated that α‐caryophyllene suppressed the growth of transplanted tumors in nude mice.

### 3.8 H&E and TUNEL Staining to Detect Apoptosis of Transplanted Tumor Tissue Cells

**FIGURE 9:**
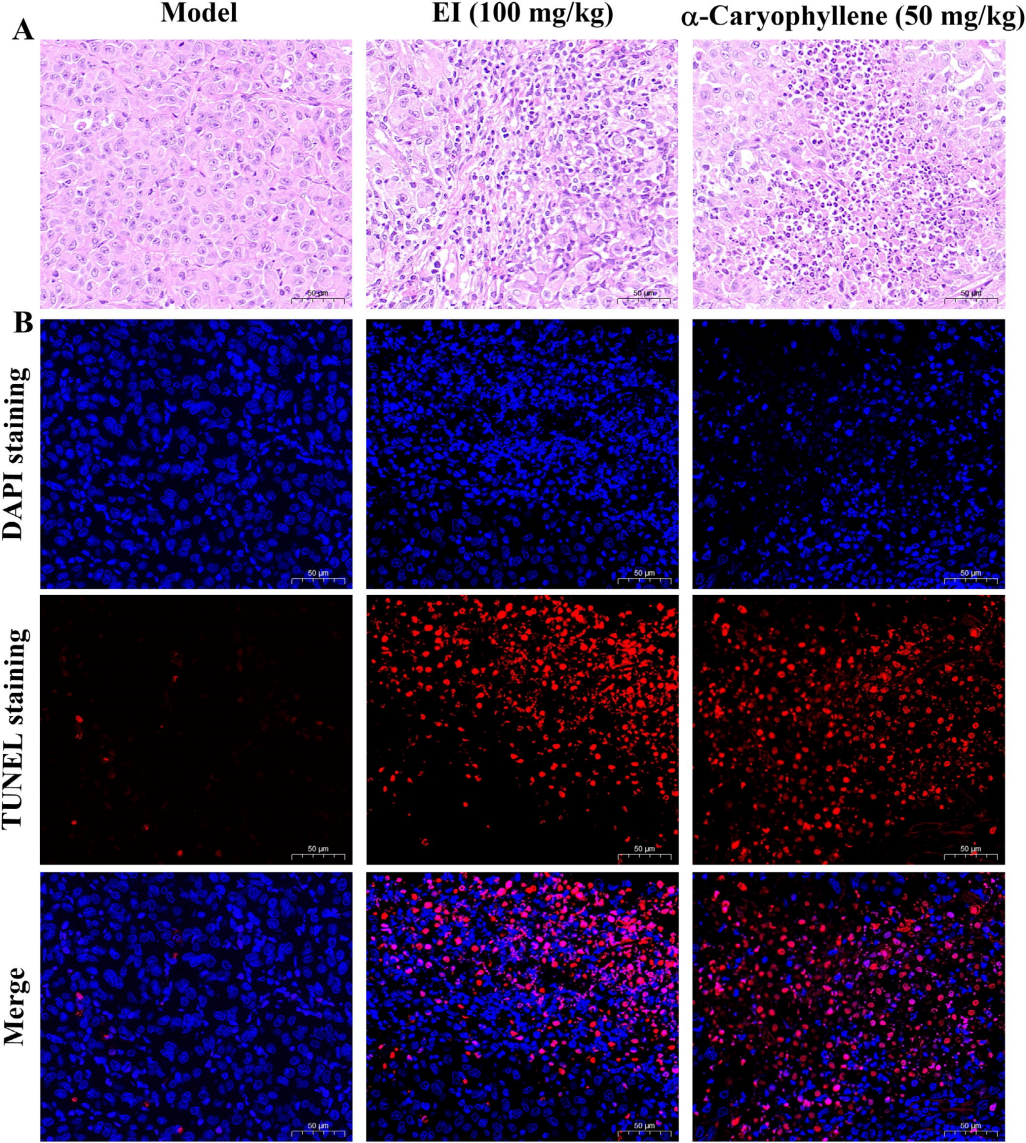
H&E staining and TUNEL staining of transplanted tumors. (A) The histomorphology of A549 transplanted tumors in nude mice was observed using H&E staining. (B) Apoptosis of transplanted tumor cells was detected using TUNEL staining.

H&E staining of transplanted tumor tissues is illustrated in Figure 9A. In comparison with the model group, the quantity of cells with cell shrinking and pyknotic nuclei in the transplanted tumor tissues of the EI (100 mg/kg) and the α‐caryophyllene group (50 mg/kg) significantly increased, and their cells were loosely arranged with larger intercellular spaces. TUNEL detected apoptosis by in situ labeling of the 3′‐OH ends generated by DNA breaks in the nucleus, which is an intuitive apoptosis detection method (Gavrieli et al. 1992). As shown in Figure 9B, the percentage of apoptotic cells showing red fluorescence was obviously increased in the EI group (100 mg/kg) and α‐caryophyllene group (50 mg/kg) compared with the model group. The above results indicated that α‐caryophyllene induced tumor cell apoptosis, thereby inhibiting the growth of transplanted A549 tumors in nude mice.

## 4 Discussion

The most crucial factor in anticancer drug screening is low toxicity to noncancerous cells and selective cytotoxicity to cancer cells (Jaudan et al. 2018). In the MTT assay, we evaluated the cytotoxic activity of α‐caryophyllene with elemene injection (EI) and cisplatin as positive control drugs. We discovered that α‐caryophyllene displayed selective cytotoxicity, which showed excellent cytotoxicity against lung cancer A549 cells and weaker cytotoxicity against noncancerous MRC‐5 and L929 cells. Notably, the cytotoxicity of α‐caryophyllene on A549 cells was better than that of EI, and its cytotoxicity on noncancerous cells was much weaker than that of EI. Past research has indicated that α‐caryophyllene exhibited selective cytotoxicity against liver cancer cells with excellent cytotoxicity against liver cancer Hep3B (IC_50_: 13.78 ± 1.46 μg/mL), HepG2 (IC_50_: 11.22 ± 1.25 μg/mL), SMMC‐7721 (IC_50_: 17.31 ± 2.03 μg/mL), and Huh7 (IC_50_: 15.09 ± 1.84 μg/mL) cells and comparatively weaker cytotoxicity against liver normal L‐02 cells (IC_50_: 115.69 ± 3.52 μg/mL) (Chen et al. 2019).

Disordered cell cycle is the major reason for the uncontrolled proliferation of cancer cells (Mishra et al. 2014). Our findings of cell cycle and clone formation assays showed that α‐caryophyllene suppressed the proliferation of A549 cells via arresting the cell cycle in the G1 phase. Cyclin‐dependent kinase (CDK) acts as a critical regulator of cell cycle progression, and CDK activity is controlled by cyclins (Mens and Ghanbari 2018; Jin et al. 2017). Prior research has demonstrated that cyclin E and CDK2 collaborate to promote the G1/S phase transition, and cyclin D binds to CDK4 and CDK6 to facilitate the G1 phase process (Niu et al. 2020; Sheng et al. 2020). Increased expression of p21 (a CDK inhibitor) inhibited G1 phase cell cycle progression (Yamauchi et al. 2017; Ge et al. 2017). According to our findings, α‐caryophyllene triggered A549 cell cycle arrest in the G1 phase via reducing cyclin E2‐CDK2 and cyclin D3‐CDK 4/6 complexes and increasing p21. Similarly, previous studies have demonstrated that α‐caryophyllene exerted antiproliferative effects in hepatocellular carcinoma HepG2 and Hep3B cells by increasing p21 and decreasing cyclin D (Chen et al. 2019).

Evasion of cell death is a distinguishing feature of tumors (Yang et al. 2024). In our research, A549 cell morphology and nuclear morphology were observed, and results showed that the cells treated with α‐caryophyllene exhibited typical apoptotic morphological alterations, like nuclear condensation, cell rounding, and cell shrinkage. In addition, the data obtained from flow cytometry analysis further confirmed that α‐caryophyllene caused apoptosis of A549 cells in a dose‐dependent manner. The Bcl‐2 protein family includes proapoptotic and antiapoptotic proteins, and apoptosis was triggered by elevating the ratio of proapoptotic protein and antiapoptotic protein, like Bax/Bcl‐2 (Dos Anjos et al. 2019; Siddiqui et al. 2015). When the Bax/Bcl‐2 ratio increases, Bax accumulates on the mitochondrial membrane, causing the ΔΨm decrease, thereby releasing Cyt c into the cytoplasm, triggering mitochondria‐mediated apoptosis (Du et al. 2023; Hou et al. 2020). The release of Cyt c into the cytoplasm caused a cascade reaction of caspase 9 and caspase 3, which led to the cleavage of PARP and induction of cell apoptosis (Liu et al. 2019). From the JC‐1 staining result, α‐caryophyllene reduced the ΔΨm in A549 cells, which indicated that α‐caryophyllene triggered A549 cell apoptosis via the mitochondrial apoptosis pathway. We further examined the levels of mitochondrial apoptosis pathway‐related proteins. Western blot results showed that α‐caryophyllene elevated the Bax/Bcl‐2 ratio, reduced ΔΨm, and promoted the release of Cyt c into the cytoplasm, thereby cleaving and activating caspase 9 and caspase 3, leading to PARP cleavage and cell apoptosis. The aforementioned findings indicated that α‐caryophyllene triggered apoptosis in A549 cells by the mitochondrial pathway. In previous studies, α‐caryophyllene induced apoptosis in liver cancer cells by promoting caspase 3 activation and PARP cleavage (Chen et al. 2019). In addition, in human pancreatic cancer cells, α‐caryophyllene reduced Bcl‐2 and increased Bax and caspase‐3 levels, exerting an apoptosis‐inducing effect (Kang et al. 2022).

The main cause of cancer death is cancer cell invasion and migration (Luanpitpong et al. 2010). MMP‐2, a matrix metalloproteinase, degrades the main component of the basement membrane (type IV collagen), thereby promoting the metastasis of cancer cells (Ricci et al. 2015; Zong et al. 2009). Highly expressed N‐cadherin promoted the metastasis of tumor cells, so lowering the N‐cadherin expression inhibited the cancer cell metastasis (Gravdal et al. 2007; Mariotti et al. 2007). Our results showed that α‐caryophyllene downregulated N‐cadherin and MMP‐2, thereby limiting the migratory and invasive capabilities of A549 cells. Previous studies have shown that α‐caryophyllene diminished colorectal cancer HT‐29 cell metastasis via upregulation of E‐cadherin (Hanušová et al. 2015).

**FIGURE 10:**
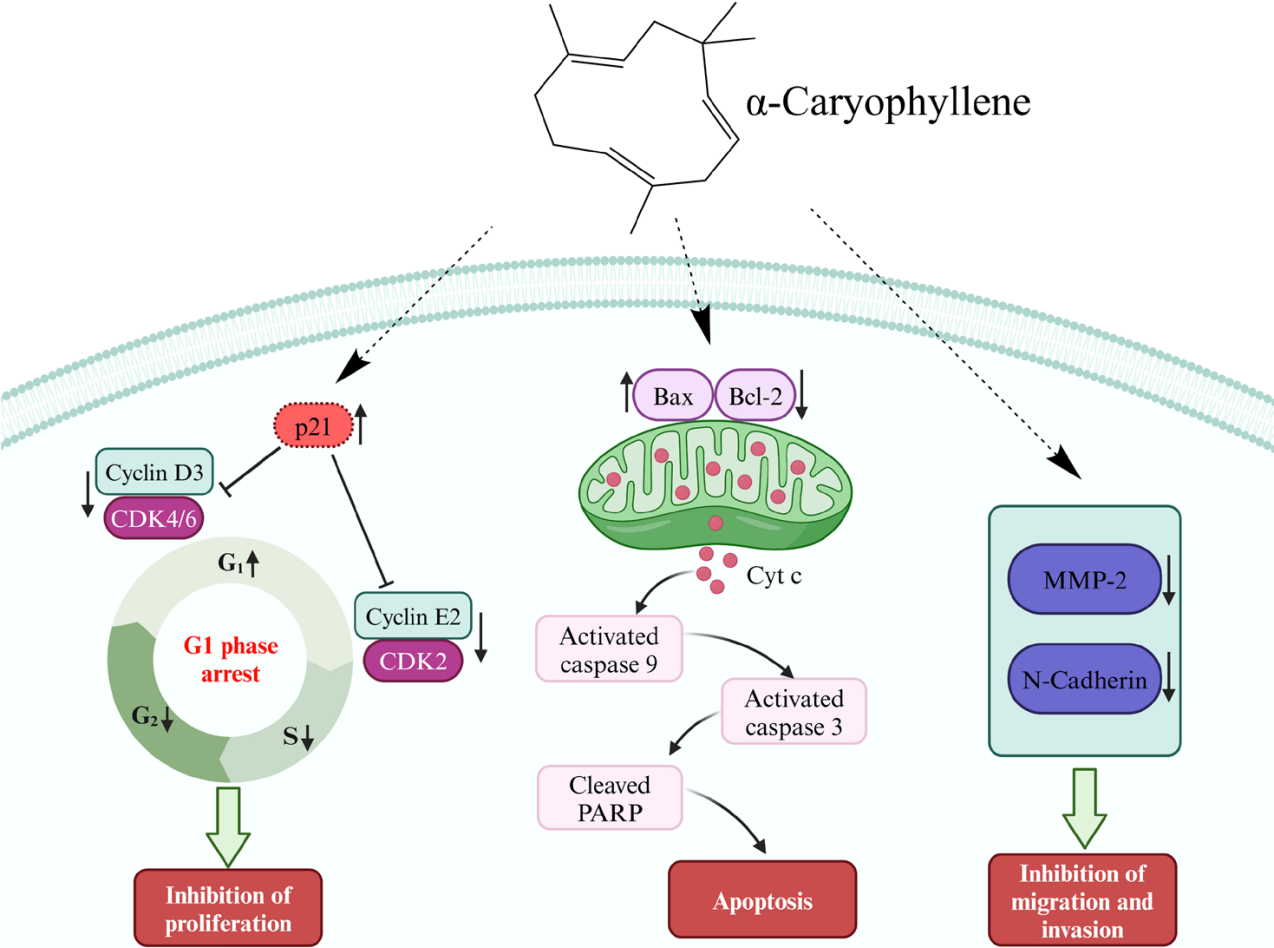
α‐Caryophyllene inhibited A549 cell proliferation, induced apoptosis, and suppressed migration and invasion.

In summary, the above studies demonstrated that α‐caryophyllene had notable anti‐lung cancer activity with anti‐proliferation, apoptosis‐inducing, and anti‐metastasis effects in vitro. As shown in Figure 10, α‐caryophyllene blocked the cell cycle in the G1 phase by reducing cyclin E2‐CDK2 and cyclin D3‐CDK 4/6 complexes and increasing p21, thereby limiting the proliferation of A549 cells. At the same time, it also activated the mitochondrial apoptotic pathway by elevating the Bax/Bcl‐2 ratio, lowering ΔΨm, releasing Cyt c, and activating caspase 9 and caspase 3, leading to PARP fragmentation. In addition, α‐caryophyllene prevented the migration and invasion of A549 cells by downregulating N‐cadherin and MMP‐2.

The nude mouse xenograft tumor test is an excellent model for exploring anticancer effects in vivo (Su et al. 2018). Therefore, we used athymic nude mice to construct a lung cancer (A549) xenograft model to further investigate the anticancer effect of α‐caryophyllene in vivo. By analyzing the volume, weight, and tumor inhibition rate of the transplanted tumor, we found that α‐caryophyllene markedly inhibited the growth of A549 transplanted tumors. In addition, we performed TUNEL staining and H&E staining on the transplanted tumor tissues. The results indicated that α‐caryophyllene suppressed the growth of transplanted tumors in nude mice via inducing apoptosis of A549 transplanted tumor cells. In in vitro MTT test results, we found that the cytotoxicity of α‐caryophyllene was better than the positive control EI. Meanwhile, the tumor inhibition effect of α‐caryophyllene administration (50 mg/kg) was comparable to that of EI administration (100 mg/kg), indicating that the anti‐lung cancer efficacy of α‐caryophyllene was still superior to the positive control EI in vivo, which was in accordance with the MTT results. Previous studies have also found that α‐caryophyllene inhibited the growth of liver cancer cell and pancreatic cancer cell transplant tumors (Chen et al. 2019; Kang et al. 2022).

Collectively, the above data demonstrated that α‐caryophyllene suppressed the proliferation of A549 cells, induced apoptosis via the mitochondrial pathway, and restricted cell metastasis in vitro; besides, it repressed the growth of A549 transplantation tumors by causing apoptosis of tumor cells in vivo. Interestingly, the anti‐lung cancer efficacy of α‐caryophyllene was superior to that of the positive control EI both in vitro and in vivo. These results provide a basis for the future development of α‐caryophyllene as a potential anticancer agent.

## 5 Conclusion

α‐Caryophyllene is a natural condiment and flavoring additive. Herein, our study is the first to report the anti‐lung cancer efficacy of α‐caryophyllene in vivo and in vitro and its potential mechanism. In antitumor activity in vitro, α‐caryophyllene exhibited obvious selective cytotoxicity on lung cancer A549 cells with lower toxicity on normal L929 and MRC‐5 cell lines, and its cytotoxicity against A549 cells was superior to that of the positive control EI. In addition, α‐caryophyllene inhibited proliferation by triggering G1 phase cell cycle arrest, triggering apoptosis via mitochondria‐mediated pathways, and inhibiting the migration and invasion of A549 cells. Meanwhile, α‐caryophyllene effectively suppressed the growth of transplanted tumors by inducing apoptosis of tumor cells, and its efficacy was superior to the positive control EI. Therefore, α‐caryophyllene has outstanding anticancer properties in vivo and in vitro with great potential to become a new anticancer drug in the pharmaceutical field.

## Author Contributions

**Lu Jin:** investigation (equal), methodology (equal), writing – original draft (equal). **Yuanquan Ran:** investigation (equal), methodology (equal). **Nian Yang:** investigation (equal), methodology (equal). **Lanlan Yang:** investigation (equal), methodology (equal). **Xiaoyan Jia:** investigation (equal), methodology (equal). **Huan Zhao:** resources (equal), validation (equal). **Qiong Hu:** validation (equal). **Bing Yang:** validation (equal). **Dongxin Tang:** conceptualization (equal), funding acquisition (equal), writing – review and editing (equal). **Minyi Tian:** funding acquisition (equal), methodology (equal), supervision (equal), validation (equal), writing – original draft (equal), writing – review and editing (equal).

## Conflicts of Interest

The authors declare no conflicts of interest.

## Data Availability

The data are available from the corresponding author upon reasonable request.
